# Multiphoton microscopy is a nondestructive label-free approach to investigate the 3D structure of gas cell walls in bread dough

**DOI:** 10.1038/s41598-023-39797-w

**Published:** 2023-08-26

**Authors:** Nanci Castanha, Sylvain Challois, David Grenier, Patricia Le-Bail, Laurence Dubreil, Tiphaine Lucas

**Affiliations:** 1 INRAE, UR OPAALE, 35044 Rennes, France; 2 INRAE, UR1268 Biopolymers Interactions Assemblies, BP 71627, 44316 Nantes, France; 3 ONIRIS, INRAE, APEX PAnTher, 44307 Nantes, France

**Keywords:** Multiphoton microscopy, Polysaccharides

## Abstract

During the different steps of bread-making, changes in the microstructure of the dough, particularly in the gas cell walls (GCW), have a major influence on the final bread crumb texture. Investigation of the spatial conformation of GCWs is still a challenge because it requires both high resolutions and 3D depth imaging. The originality of the present work lies in the use of label-free non-destructive multiphoton microscopy (NLOM) to image the 3D structure of GCWs, shedding light on their behavior and organization in wheat bread dough. We demonstrated that second and third harmonic generation (SHG, THG) allow imaging, respectively, of starch granules and interfaces in bread dough, while the gluten matrix was detected via two-photon excitation fluorescence (TPEF). Last, a distinction between the gluten network and starch granules was achieved using gluten endogenous fluorescence (EF) imaging, while the position, size, and 3D orientation of starch granules in GCWs were determined from harmonic imaging, made possible by the acquisition of backward and forward SHG with linear polarization. These innovative experiments highlight the strengths of NLOM for a label-free characterization of bread dough microstructure for the first time, in order to understand the role of starch granules in dough stabilization.

## Introduction

In order to better understand and optimize the macroscopic behavior of bread to improve its quality, it is essential to evaluate the microstructure of the dough—in other words, the microscopic organization of its major constitutive components: gluten, starch, and gas cells—as well as the changes it undergoes during the different processing steps of breadmaking^1^. To this end, a variety of microscopy and imaging techniques have been applied over past decades to provide a picture of bread dough microstructure.

Conventional light microscopy (LM), for example, was used to describe the organization and development of different components in bread dough before and after baking. In a pioneering study, Burhans and Clapp^2^ evaluated bread dough microstructure from mixing to baking using LM. Just over a decade later, Sandstedt et al.^3^ combined LM with staining of the gluten network, revealing the organization of the gas cell walls (GCWs) in bread dough just before and after baking. Thus far, this is the only set of images available in the literature for thin GCWs (with just one or two rows of starch granules in alignment). Huang and Moss^4^ also combined LM and protein staining to describe the mechanisms involved in the development of the gluten network in steamed bread dough. Hug-Iten et al.^5^, instead of staining proteins, combined LM and staining of the starch granules (using iodine vapor) to qualitatively evaluate their morphology in dough and in fresh and aged breadcrumbs. There are many other examples of the use of such techniques. However, despite all care being taken in sample preparation, the fixation, dehydration, and/or staining steps can interfere with a sample’s microstructure, leading to misinterpretation of the images^3, 5–7^.

Electron microscopy techniques, such as scanning electron microscopy (SEM) and transmission electron microscopy (TEM), have also been widely applied in bread science because of better imaging resolution compared with LM^8^. Amend and Belitz^8–10^, for instance, published several works using electron microscopy in breadmaking, thoroughly evaluating the spatial organization of gluten. In turn, Roman-Gutierrez et al.^11^ used SEM and environmental SEM (ESEM) to reveal the initial microstructure of different wheat flours and their main components (starch, gluten and pentosans), as well as their changes during hydration. In a more recent study, Yang et al.^12^ combined SEM with several other analytical methods to suggest that starch played a more decisive role in mixing properties than gluten. In all cases, preparation of the samples (through dehydration and sectioning, for example) and the lack of staining to differentiate the components, especially for complex compositions as in bread dough, present major obstacles to establishing firm conclusions from electron microscopic observations^9, 11^.

One alternative is to use Confocal Laser Scanning Microscopy (CLSM) with appropriate staining (applying fluorochromes with specific excitation spectra, often in combination) to visualize bread dough microstructure with good imaging resolution. This method offers the possibility of depth imaging and 3D reconstruction of the sample—although this last feature has rarely been exploited^13^. Turbin-Orger et al.^14^, or Dubreil et al.^15^, for example, used CLSM combined with multilabeling to identify lipids and proteins in the liquid films that separate the gas cells in wheat flour dough, or gas cells, lipids, and puroindoline-a simultaneously in bulk dough. Quantitative analysis has also been conducted on CLSM images with specific staining. Bousquieres et al.^16^, for example, quantitatively evaluated laminated dough to differentiate fat from dough layers while Jekle and Becker^1^ evaluated the effects of added water on the microstructure of dough proteins, characterized by average size, area fraction and perimeter, among others. It is important to mention that, despite some pioneering studies, CLSM images of dough in the literature are mainly the object of qualitative evaluation. Once again, sample preparation can be a drawback^7, 17^.

Despite the various imaging techniques available, the literature review revealed that the majority of studies on dough microscopy have focused on bulk dough at the mixing or shaping steps (dough development). Very few studies focus on the latter stages of breadmaking, where the dough has been stretched between bubbles of increasing size, despite the fact that the degree of extension and the moment of GCW rupture are known to be crucial for good aeration and fine texture in crumb^18, 19^. Conceptual schemes for phase organization in GCWs at these advanced processing stages have been proposed by some authors^18–21^, relying on the small number of microscopic images of thin GCWs available until now^3, 14^. In addition, the exact role played by starch granules in GCW (de)stabilization is not yet clear, while we do know that GCW mechanical properties partially depend on the proportion and location of starch granules in the GCW^19^. More information could be acquired by microscopy, ideally with 3D exploration given the geometry of these granules. It also seems clear from the review above that, to gain a more accurate visualization of the spatial organization of the dough’s main components (especially starch) in GCWs, a microscopic technique that combines good image resolution with minimal sample preparation and a label-free approach is desirable. Raman microscopy and non-linear optical microscopy (NLOM) are good candidates in this regard.

Huen et al.^22^ have already applied Raman spectroscopy to mixed dough at the frozen state, with the objective of disentangling the signals from ice, liquid water, starch, gluten and yeast with the aid of reference spectra for these pure components. NLOM, for its part, has never been applied in dough evaluation before, despite having been successfully used to image a large range of label-free biological samples^23^ using Second Harmonic Generation (SHG) and Third Harmonic Generation (THG) contrast exploration. Use of SHG from collagen or myosin bands in muscle^24–26^ and from amylopectin in starch granules^27, 28^ has been described in the literature. Furthermore, SHG was used to image starch granules in plants^29, 30^ and starchy food products^6, 31^, with promising results. THG from local transitions in the refractive index produces contrasts that can be used to explore heterogeneity in materials and the interfaces between phases in particular^32, 33^. Thus, harmonic microscopy has the potential to be a valuable technique in evaluating label-free bread dough, with SHG imaging of the starch granules and THG imaging of the starch-dough-gas interfaces. Last, with regard to 3D analysis, NLOM is also advantageous in terms of penetration depth, since the infrared excitation wavelengths reduce light scattering and sample absorption^34, 35^.

In the present work, an innovative approach based on non-destructive NLOM was applied to label-free proven dough. The objective of this pioneering study was to analyze the spatial dispersion of phases in proven dough, especially starch granules and gluten network in GCWs, and also examine the morphology of the GCW envelope and these embedded starch granules, providing new microscopic information to support the hypotheses about the role played by starch granules in GCW (de)stabilization. To that end, we developed and assessed a non-destructive multiphoton microscopic approach, compatible with the bio-distribution analysis of the starch granules in GCWs at different stages of extension, using both two-photon excitation fluorescence (TPEF mode) and harmonic generation signals (SHG, THG) obtained from the label-free dough. The potential for a depth-based (3D) exploration of dough microstructure at different scales (that of gas cells and that of GCWs) under near-infrared multiphoton excitation was further evaluated. This was the first use of NLOM to image dough and GCWs, and has demonstrated its potential to expand knowledge in the breadmaking field.

## Results

Firstly, a multimodal study of label-free dough was performed by combining THG, SHG and endogenous fluorescence (EF) acquired using NLOM, to better explore the heterogeneity of this material and the interfaces between its components. Despite promising results, the evaluation of the 3D organization of the GCWs was impaired by the THG mode (as better discussed below). After some further adaptations, SHG and EF were combined to image a number of thin GCWs, evaluate their 3D organization, with respect to starch granules in particular, and provide information on some of the morphological features of these starch granules.

A number of different typical image stacks acquired from proven dough are described in this section to illustrate the exploration pathway and of its major outcomes. Table 1 gives an overview of their acquisition parameters. In all cases, dough samples were analyzed at the end of proving to ensure maximum stability during acquisition.

**Table 1:** Settings for acquisition parameters and size of NLOM images in Figs. 1, 2, 3, 4, 5 and 6.

Figure number/sample		Excitation wavelength (nm)	Laser power (mW)	Signal detection in separate channels (nm)				Number of images (step plane)	Pixel size (µm)	Frame averaging	Scan speed (frames/s)	Acquisition time (min)	Size of image (X × Y × Z)
				Blue GaAsP	Green GaAsP	Yellow GaAsP	Red PMT						
Figure 1		1240 (THG and SHG red) 1040 (EF and SHG green)	734 (1240 THG), 642 (1240 B-SHG), 24 (1040 B-SHG), 126 (1040 EF)	415/10 (B-THG)	515/20 (B-SHG)	575/25 (B-EF)	629/56 (B-SHG)	46 images (0.5 µm)	0.17	2	0.25 (galvanometric scanner)	12.27	177.36 × 177.36 × 22.5 µm
Figure 2		1240 (THG and SHG red) 1040 (EF and SHG green)	570 (1240 THG), 570 (1240 B-SHG), 123 (1040 B-SHG), 123 (1040 EF)	415/10 (B-THG)	515/20 (B-SHG)	575/25 (B-EF)	629/56 (B-SHG)	47 images (0.5 µm)	0.17	2	0.25 (galvanometric scanner)	12.53	177.57 × 177.36 × 23.0 µm
Figure 3		820	93 (820 SHG), 169 (820 EF)	415/10 (B-SHG, F-SHG)	525/50 (EF)	Not used	Not used	10 images (0.5 µm)	0.17	32	15 (resonant scanner)	2.84	99.91 × 100.08 × 4.5 µm
Figure 4		820	93 (820 SHG), 169 (820 EF)	415/10 (B-SHG, F-SHG)	525/50 (B-EF)	Not used	Not used	1 image	0.51	32	15 (resonant scanner)	0.28	527.35 × 527.43 × 0 µm
Figures 5 and 6	GCW 1	820	93 (820 SHG), 240 (820 EF)	415/10 (B-SHG, F-SHG)	525/50 (B-EF)	Not used	Not used	84 images (0.5 µm)	0.17	16	15 (resonant scanner)	11.95	175.54 × 175.78 × 41.5 µm
	GCW 2	820	93 (820 SHG), 240 (820 EF)	415/10 (B-SHG, F-SHG)	525/50 (B-EF)	Not used	Not used	73 images (0.5 µm)	0.17	16	15 (resonant scanner)	10.38	175.53 × 175.53 × 36.0 µm
	GCW 3	820	93 (820 SHG), 240 (820 EF)	415/10 (B-SHG, F-SHG)	525/50 (B-EF)	Not used	Not used	48 images (0.5 µm)	0.17	16	15 (resonant scanner)	6.83	175.78 × 175.78 × 23.5 µm
	GCW 4	820	93 (820 SHG), 240 (820 EF)	415/10 (B-SHG, F-SHG)	525/50 (B-EF)	Not used	Not used	93 images (0.5 µm)	0.17	16	15 (resonant scanner)	13.23	175.78 × 175.78 × 46.0 µm

*SHG* second harmonic generation, *THG* third harmonic generation, *EF* endogenous fluorescence, *GaAsP* gallium arsenide phosphide detector, *PMT* photomultiplier tube detector, *B* backward, *F* forward.

**Figure 1:**
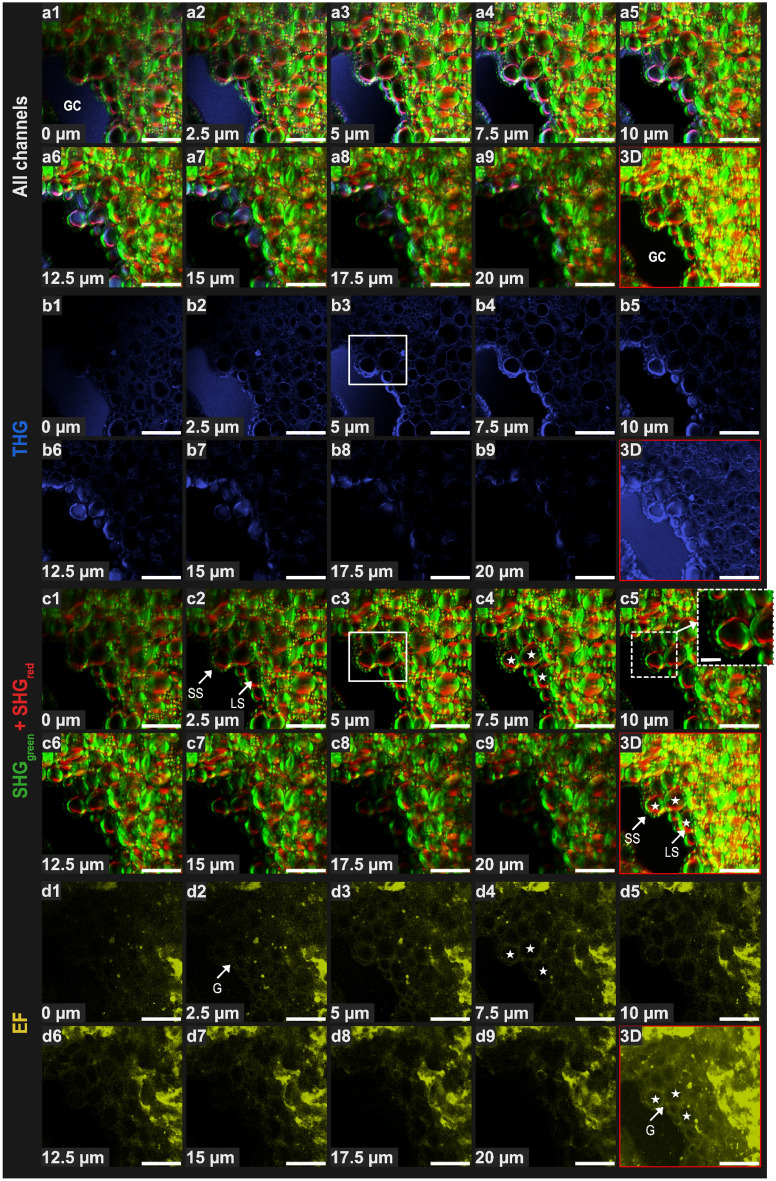
Second harmonic generation (SHG) and third harmonic generation (THG) combined with endogenous fluorescence (EF) to image starch granules, gluten network, and gas-dough interface in label-free bread dough (bulk) at the end of proving, observed at different depths (z-stack 20 µm, 9 images every 2.5 µm) and in 3D representation for each channel (red square). (**a**) Combination of the three modes, all channels (THG, SHG, EF); (**b**) THG in blue (1240 nm); (**c**) SHG in green and red under dual excitation: 1040 nm (green)/1240 nm (red); (**d**) EF in yellow (1040 nm). *G* gluten, *GC* gas cell, *LS* large starch granules, *SS* small starch granules. See text for information on white squares in images (**b3,c3**) and white stars in images (**c4,d4**) and 3D representations of SHG and EF channels. Scale bars = 50 µm (original size); 12.5 µm (inset area—dotted white square).

**Figure 2:**
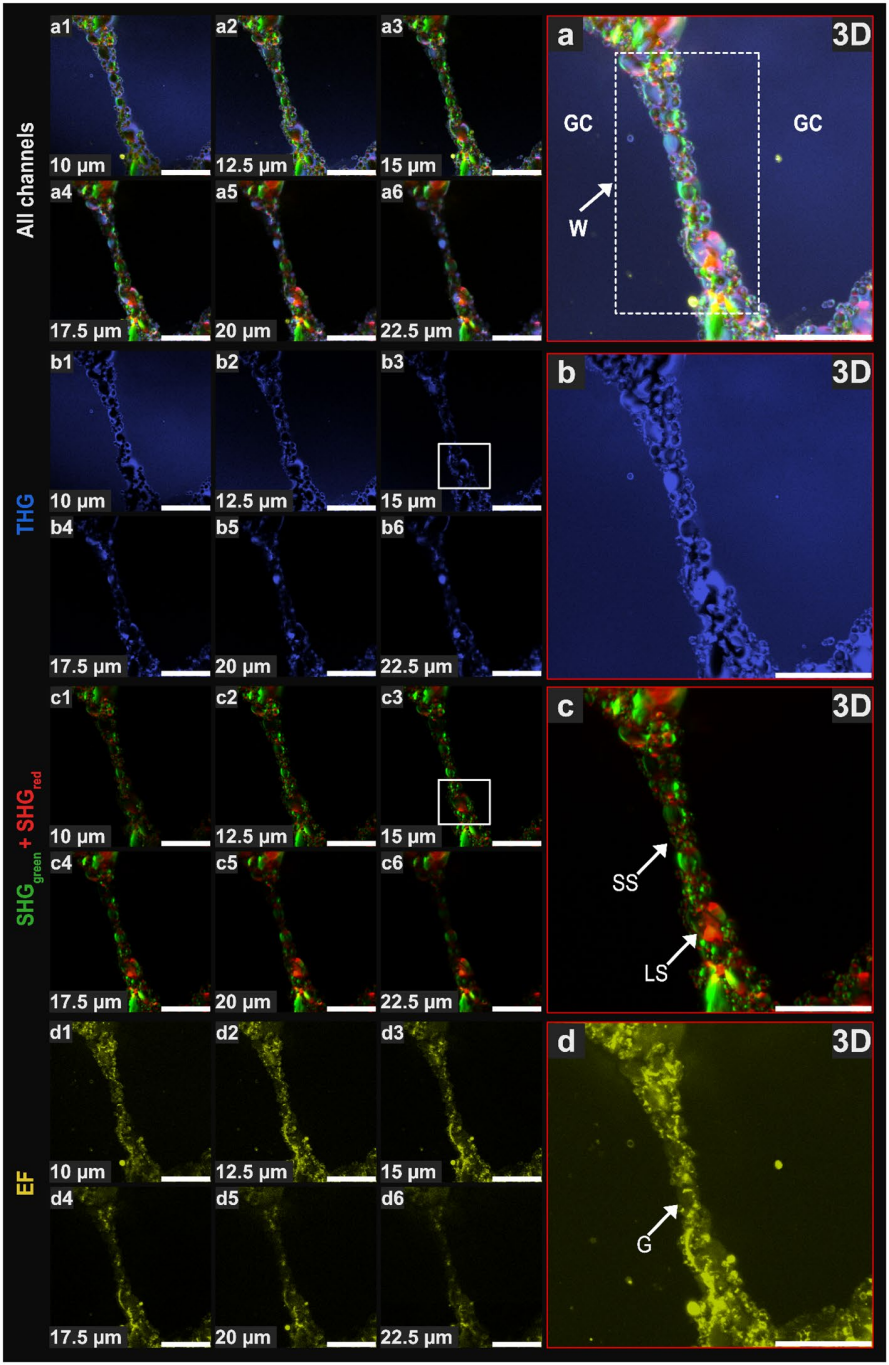
A typical thin gas cell wall (GCW) from label-free dough at the end of proving analyzed by NLOM, using harmonic and fluorescence acquisitions, and observed at different depths (z-stack from 10 to 22.5 µm, 6 images every 2.5 µm) with a 3D representation for each channel (red square). (**a**) Combination of the three modes, all channels (THG, SHG, EF); (**b**) THG in blue (1240 nm); (**c**) SHG in green and red under dual excitation: 1040 nm (green)/1240 nm (red); (**d**) EF in yellow (1040 nm). *G* gluten, *GC* gas cell, *LS* large starch granules, *SS* small starch granules, *W* gas cell wall. See text for information on white squares in images (**b3,c3**). Scale bars = 50 µm.

**Figure 3:**
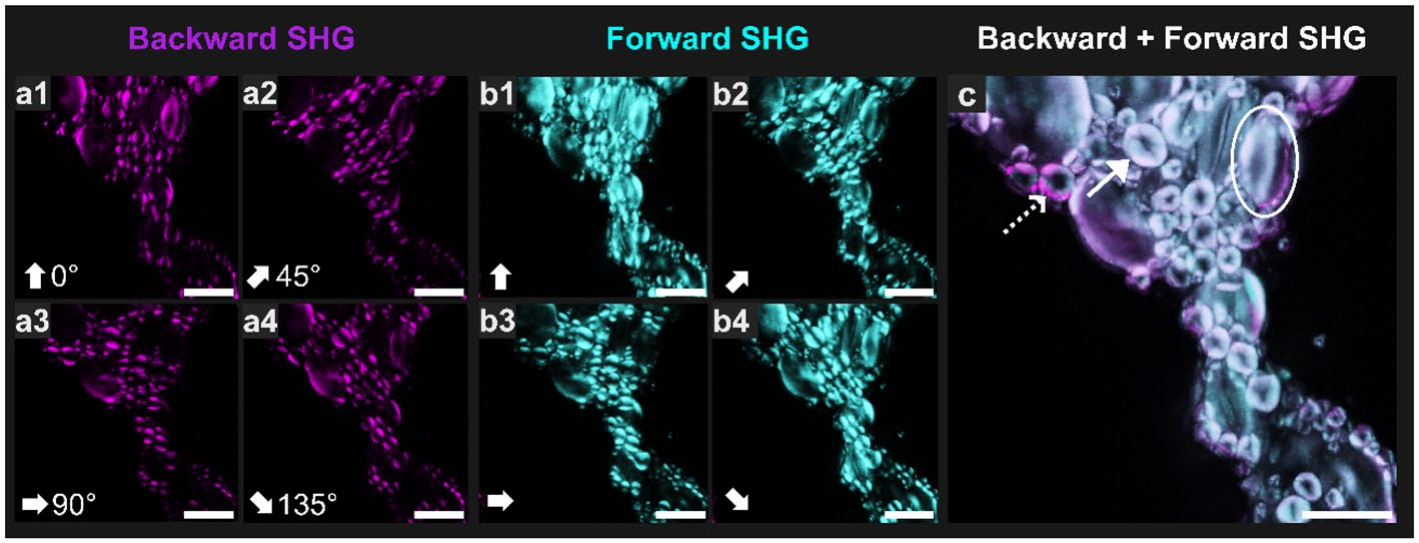
Backward- and Forward-SHG (B- and F-SHG) imaging of starch granules in bread dough using linear polarization states corresponding to 0°, 45°, 90° and 135° angles (white arrows in the lower left-handed corner of each image indicate the direction for that image). (**a**) B-SHG; (**b**) F-SHG; (**c**) combination of B- and F-SHG and of all polarization states. See text for explanation of white arrows and white ellipse in image (**c**). Scale bars = 10 µm.

**Figure 4:**
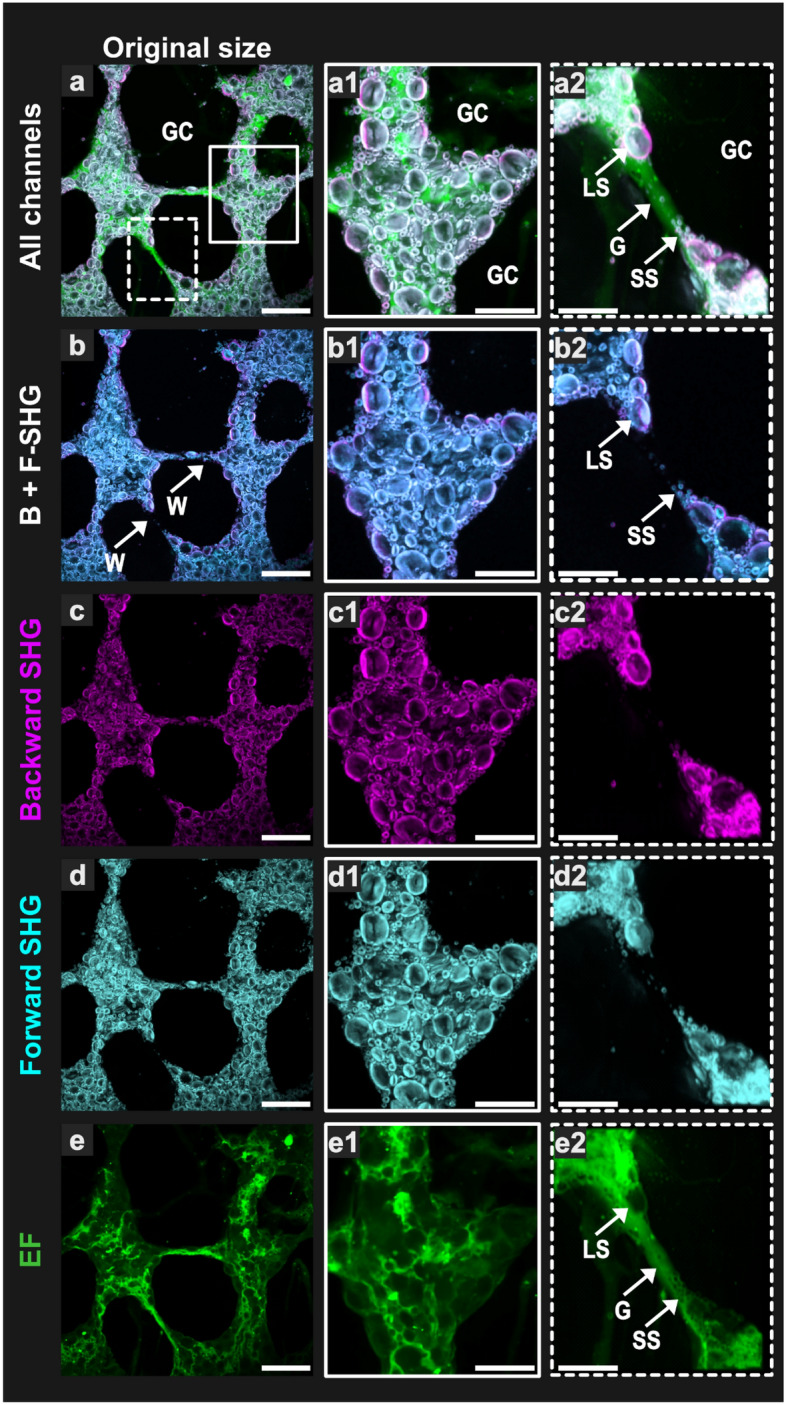
Combination of EF and polarized backward- and forward-SHG to image bread dough with all its constitutive phases at the end of proving. First column, original size images. Second column, first Region of Interest (ROI) delimited by the white square in image (**a**). Third column, second ROI delimited by the dotted white square in (**a**). For SHG, the four polarization angles were combined. *G* gluten, *GC* gas cell, *LS* large starch granules, *SS* small starch granules, *W* gas cell wall. Scale bars = 100 µm (original size image); 50 µm (ROI).

**Figure 5:**
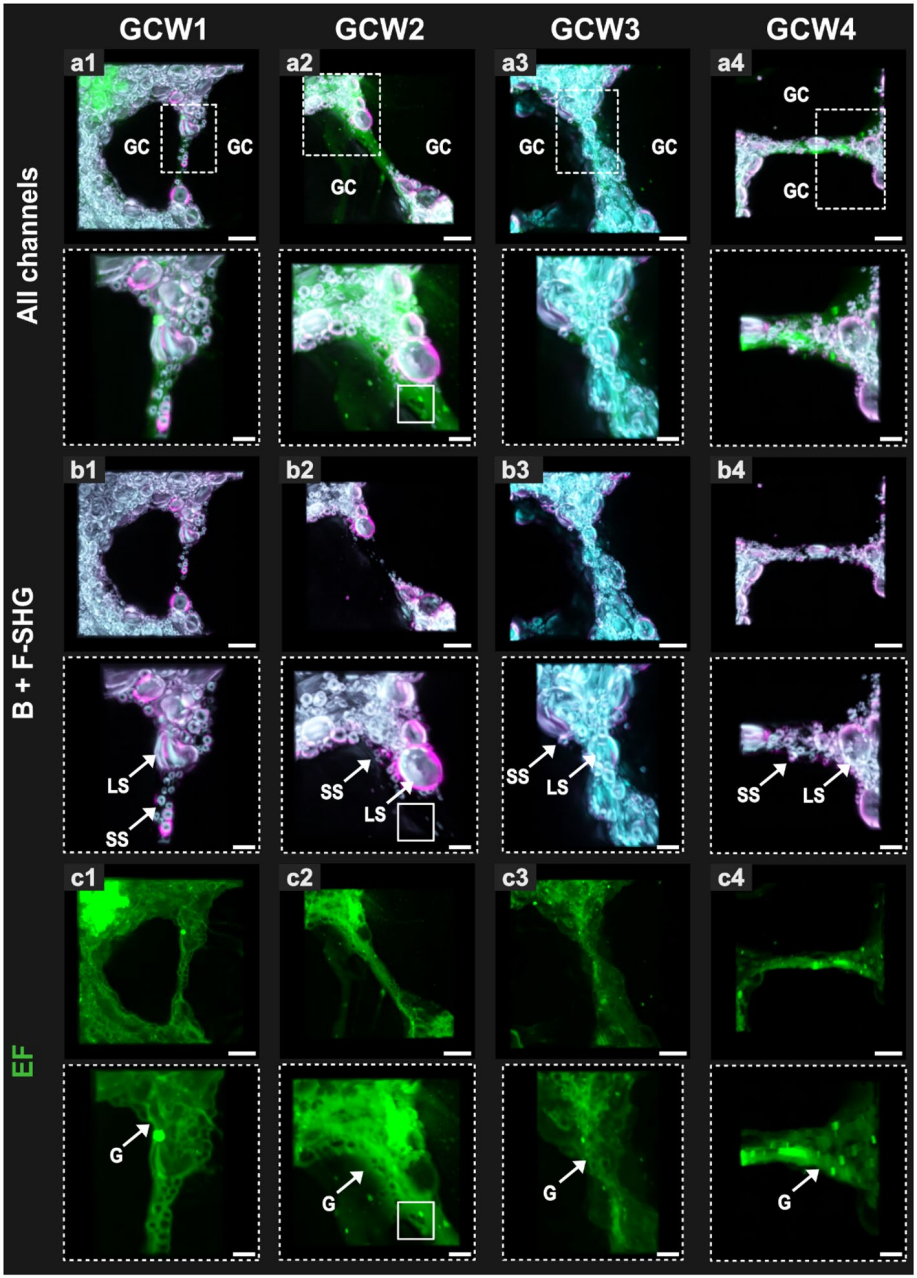
3D representation of four different GCWs imaged from bread dough at the end of proving with combined EF and polarized SHG. For each GCW, rows one (**a1–a4**), three (**b1–b4**) and five (**c1–c4**) show the original images of the GCWs in different channels, respectively: merge of all channels, Backward-SHG + Forward-SHG, and EF mode. Rows two (merge of all channels), four (Backward-SHG + Forward-SHG) and six (EF) show the regions of interest (ROI) indicated by dotted white squares in row one. The four polarized angles were combined for each image. *G* gluten, *GC* gas cell, *LS* large starch granules, *SS* small starch granules. See text for explanation of white squares in the ROI images in the second column. Scale bars = 25 µm (original size); 10 µm (ROI).

**Figure 6:**
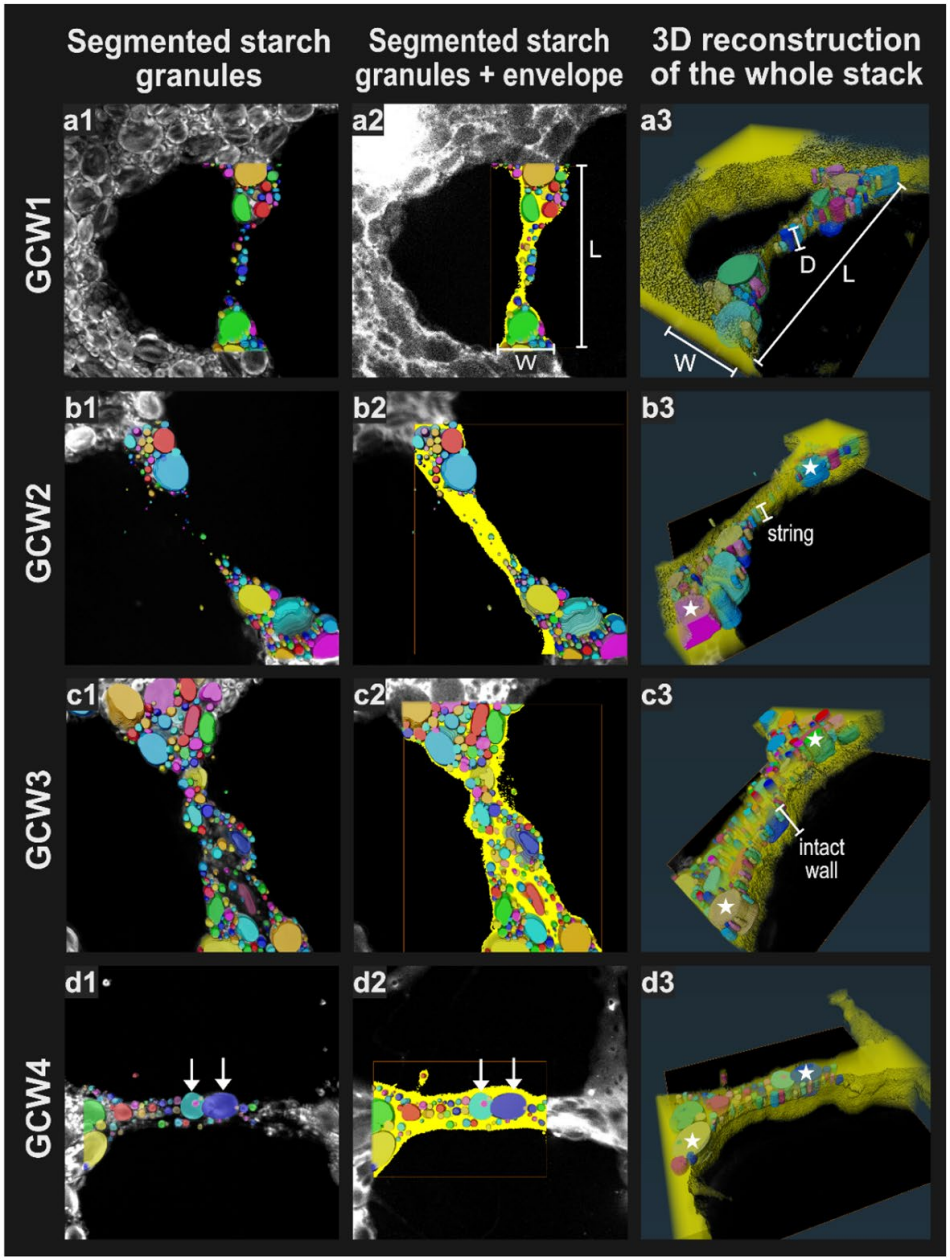
Image analysis of the GCWs shown in Fig. 5, using arbitrary colors. Column 1: 3D top-view of starch granules segmented manually with the aid of SHG contrast (image background is produced using SHG mode). Column 2: GCW envelope (in yellow) delimited using the EF signal and based on a single z-slice, so that all segmented starch granules present above the selected z-slice for the envelope are visible (background using all modes). Column 3: top view of the 3D reconstruction of the envelope and the segmented starch granules by transparency. *W* width, *L* length, *D* depth. See text for details on white arrows and stars.

### Use of second and third harmonic generation combined with endogenous fluorescence (EF) to image microstructure of bread dough

The dough phases, i.e. the aqueous granular phase (mostly composed of starch granules), the hydrated gluten network and the gaseous phase dispersed across numerous cells of increasing size, were imaged using a multimodal and dual excitation approach (1240 nm/1040 nm) to acquire EF, THG and SHG combined signals, with backward mode detection. THG was acquired in the blue channel (415/10 nm, excitation 1240 nm), EF was acquired in the yellow channel (575/25 nm, excitation 1040 nm) and SHG was acquired in the remaining two available channels: green (515/20 nm, excitation 1040 nm) and red channels (629/56 nm, excitation 1240 nm) (Table 1). Typical images obtained with this protocol are illustrated in Fig. 1 for bulk dough and in Fig. 2 for a GCW. The figures show different penetration depths (images acquired every 2.5 µm) and one 3D representation (square outlined in red) for each channel.

Combined harmonic and fluorescence acquisitions performed in four channels are illustrated in Figs. 1a and 2a. Sequential images (Figs. 1a1–a9, 2a1–a6) were selected at 2.5 µm intervals to show the signal evolution of the merged channels at increased depths. By producing separate images for each channel (Figs. 1b–d, 2b–d) the evolution of each signal can also be tracked by depth.

Figure 1b shows that detection through THG (blue channel) mainly occurred at the starch-dough-gas interfaces, as expected. THG showed clear outlines for the starch granules at shallow depths within the sample (Fig. 1b3,b4, for instance). However, the THG signal was shown to vary with depth, being lost progressively from a depth of 10 µm and completely at 20 µm (Fig. 1b9), whereas SHG (Fig. 1c9) and EF (Fig. 1d9) signals were detected at this depth. Furthermore, although THG was high at the gas/dough interface, it also formed a halo that hindered visualization of the components closest to the interface, including very small starch granules (compare white squares in Fig. 1b3,c3: these granules are revealed by SHG in c3, discussed further below, but are merged in b3). Because the intensity of this halo was also dependent on depth, only the largest granules located at the gas/dough interface were revealed, thereby making this interface appear discontinuous (Fig. 1b5–b9). Similar behavior was observed for the THG signal produced by the GCW (Fig. 2b), with a loss of depth contrast (Fig. 2b4–b6) and the merging of small granules located close to the interfaces (compare white squares in Fig. 2b3,c3).

The SHG signals obtained in the green and red channels (Figs. 1c, 2c) were not located in the same area of the starch granule, but instead complemented each other to produce an almost continuous outline for each granule (inset in Fig. 1c5). This was probably due to the different polarization of the two beams (1040 nm and 1240 nm). It is known that the use of different laser radiation polarization values allows imaging of the organization of semi-crystalline domains of amylopectin as concentric layers in starch granules^28^. Hence, by combining the SHG signals acquired in the green and red channels, it was possible to image amylopectin for both the minor and major axes of the starch granule. With SHG imaging, even the smallest starch granules (SS) were revealed (marked by white arrows in Figs. 1c2, 2c), especially those located at the gas/dough interface (inset, Fig. 1c5), opening up the possibility for future studies to explore the Pickering effect, a very important mechanism in GCW stabilization^36^.

The morphology of the autofluorescent area (EF) displayed a fibrillary texture as could be expected for the gluten network (more easily seen in Supplementary Material Fig. S1d). In Fig. 2d, the most intense autofluorescence was interwoven between the starch granules to a certain extent, passing from the surface of one granule to the opposite surface of the next granule. Additionally, the autofluorescence in the bulk dough outlined round shapes with low signal intensities corresponding to large granules that were visible using SHG (compare areas marked by white stars in Fig. 1c4,d4, as well as in the 3D representations of SHG and EF channels).

For the purpose of our study of thin GCWs, where the distance between the gas interfaces of the stretched dough is very small, the oversaturation of the THG signal at the gas/dough interfaces constituted a drawback for the observation of the granular phase located between them (Fig. 2b). Indeed, it was difficult to isolate and segment the starch granules individually and in 3D using the THG mode. This result does not affect the considerable potential of THG for future investigations of interfaces in bread dough where the characterization of deeper layers is not an essential factor, as can be seen in Figs. 1b and 2b.

### Combining backward-SHG and forward-SHG with linear polarization for exhaustive starch granule SHG detection

We improved the efficacy of anisotropic SHG detection by including forward–backward signal detection and using 4 polarization angles for the excitation beam. This revealed the outlines of the starch granules and facilitated their morphological analysis. The improvements brought by this acquisition protocol to the outline imaging of the starch granules are presented in Fig. 3 with a combined magnified image of a small number of starch granules.

Backward-SHG and Forward-SHG (B- and F-SHG) image acquisition performed at 0°, 45°, 90° and 135° showed polarization anisotropy of the starch granules, with mutually complementary SHG signal information detected in different areas of the granules. In each starch granule, two lobe structures appeared to be oriented parallel to the linearly-excited polarization direction (see white corner arrows in Fig. 3a,b). According to the literature, such two lobe structures are attributable to the radial arrangement of the SHG-active crystalline amylopectin in the starch granule^37^.

The summed reconstruction of linear polarization images obtained from the four linear polarization angles (Fig. 3c) made possible the outline imaging of whole starch granules in bread dough. The white ellipse in Fig. 3c, for example, shows a starch granule that is only fully observed when all the polarization angles and backward and forward detections are combined (the same granule is almost not visible in Fig. 3a3, for example). This process will facilitate the segmentation of starch granules, offering more reliable access to the morphology and size of even the smallest granules. In Fig. 3c granules are between 4 and 10 µm in size, corresponding mainly to B-type granules for wheat starch, according to the classification based on size ranges suggested by Wilson et al.^38^: A-type granules (> 15 μm), B-type granules (5–15 μm), and C-type granules (< 5 μm).

B-SHG appeared more intense at the periphery of each starch granule than in the inner part in comparison to F-SHG (Fig. 3c, dotted white arrow), as has been described elsewhere^39^. In addition, the SHG imaging of starch granules depicted black centers to the granules, as shown by the white arrows in Fig. 3c. This absence of a SHG signal in the center of starch granules has already been observed in the literature, and is thought to be due to the centrosymmetric structure of the hilum^40^.

### Proven dough microstructure imaging from polarized SHG and EF

A single excitation at 820 nm was used to acquire EF in the green channel (525/50 nm) and SHG in the blue channel (415/10 nm) (Table 1), with both backward (magenta) and forward (cyan) signal detection. In addition, by varying the incident polarization angle, it was possible to acquire an exhaustive SHG signal generated by starch granules from oriented crystalline amylopectin concentric layers. Last, fast image acquisition was made possible using a resonant scanner (15 frames/s for image 1024 × 1024), obtaining high EF and SHG signals from 820 nm excitation.

By combining the polarized-SHG protocol with the EF signal it was possible to acquire the main microstructural features of label-free dough (Fig. 4), featuring a continuous gluten network (G, marked by arrows in Fig. 4a2,e2), dispersed starch granules (LS and SS, marked by arrows in Fig. 4a2,b2,e2—appearing in the last as “empty” endogenous fluorescence objects), and gas cells, which appeared black in all modes (GC, Fig. 4a).

The dough microstructure observed using label-free NLOM was in accordance with those described in the literature using CLSM with fluorescent probes^17^ and even SEM^41^ at higher magnification. Numerous gas cells were separated by walls of variable thickness (from 5 to 50 µm, examples marked W in Fig. 4) and composed of starch granules and gluten (Fig. 4a2). This size distribution for GCWs was well in line with that reported by Turbin-Orger et al.^14^ using synchrotron X-ray tomography of dough during proving. The thinnest walls were mainly constituted of gluten (gluten film before rupture or string, see below) and a few granules, located at the edges (Fig. 4a2–e2) whereas the largest had the appearance of bulk dough, with an apparently continuous gluten network of non-uniform density containing numerous embedded starch granules (Fig. 4a1–e1).

### Gas cell wall microstructure imaging with morphometric analysis of starch granules

Different GCWs were depicted using NLOM, being acquired at distinct degrees of the dough extension, therefore presenting different configuration of thickness and shape (Fig. 5). In comparison to the previous section, note that the scale of the GC is sacrificed to achieve higher resolution in the images of the GCW (GCW2 maintains the scale of the previous analysis).

#### Dimensional and phase analysis of GCWs based on visual inspection

Before commenting on the different modes displayed in Fig. 5, it is worth providing additional information about the size of the GCWs. Lengths, widths and depths (L × W × D) were 125 × 12 × 17 µm (GCW1), 170 × 20 × 25 µm (GCW2), 160 × 34 × 41.5 µm (GCW3), and 150 × 30 × 45 µm (GCW4). More information on their measurement and the spatial baseline is provided under “Materials and methods” section. It is important to mention that these values were approximate, since a GCW’s dimensions can change depending on where the measurement was performed and the above values for width and depth were reported at the midpoint of the length of the GCWs. GCW1, for example, showed a width variation from 22 µm (where the starch granules were aligned in two rows) to 8 µm (where the gluten was visibly free of starch granules). The width at mid-length was around 12 µm (value considered). Additionally, the full depth of the image (z-stack) and the analyzable GCW depth (evaluated using the EF signal intensity) were found to be very different in some stacks. Still looking at GCW1, its depth acquired via EF (17 µm) was much less than that of the stack of images (z-stack = 41.5 µm deep, as detailed in Table 1). We can therefore consider GCW1 to be a “string”, that is, the remains of a GCW that has not fully ruptured^42^. In line with this dimensional analysis, GCW1 and GCW2 were considered to be strings (their depths were almost half the extent of their image stacks and similar to their measured widths), while GCW3 and GCW4 were considered to be intact walls (their depths were found to occupy almost the whole extent of the image stack, while their width encompassed that of several starch granules, a sign that stretching of the wall is less advanced). To illustrate this, a depth-based view of these strings and intact walls is provided in Fig. 6 (a3 to d3).

As stated above, it was possible to discern both granular and gluten phases (the latter being assigned to areas of high autofluorescence intensity) in the walls/strings studied. For the first time and with no labelling, we were able to examine a thin wall (GCW4), consisting of one row of starch granules and to detect the initiation of gluten films at two locations between these granules; it was also possible to examine longer threads of gluten containing no starch granules, deemed to be the remains of a GCW rupture in the GCW1 string. Starch granules were at their smallest within the wall; this was markedly the case for GCW3, but with some exceptions in GCW4 (see white arrows, Fig. 6d1,d2). By contrast, lenticular granules frequently presented their larger surface at the edges of the walls. The presence of very small gas bubbles ranging from 4 to 7 µm in diameter (see white squares in ROI images for GCW2, Fig. 5, second column) within the thin GCWs was suspected on the basis of comparison between SHG and EF modes (dark intensity in both modes). In fact, the thinning of large GCWs is produced by the successive rupturing of the outer walls of the many small GCs trapped in this larger wall^43^. Our observations suggested that some tiny GCs might remain even after extensive stretching of the GCW.

Following the visual examination of the different types of GCW, the next step was to further process the images through segmentation. The individual starch granules imaged using SHG were delineated from the rest of dough, and the envelope of the GCWs imaged using EF was shown separately from the background signal (gas cells), see “Results” section in Fig. 6 (first and second columns respectively). Labelling step assigns an arbitrary color to the different objects, helping to distinguish granules individually or GCW from gas. This allowed further quantification of the starch granules (see below) and also enabled a more detailed 3D reconstruction of the GCWs and the granular phase within it (last column in Fig. 6).

#### Image analysis using segmentation on images acquired by SHG and TPEF modes, separately

The segmentation of the starch granules was facilitated by (i) the specificity of SHG imaging to starch granules, with no background images, (ii) the perfect outline of the starch granule obtained through SHG polarization imaging. However, because the SHG signal decreased more strongly with depth than the EF signal, starch granule segmentation was not performed across the full depth of the stack (Fig. 6a3–d3). Once individually segmented and put into 3D perspective (Fig. 6a3–d3), the starch granules appeared well defined in the x–y axis but deformed in the z-axis, being somewhat tubular in shape rather than having the spherical or ellipsoidal form expected for wheat starch granules^38^ (white stars). This elongation of the starch granules along the z-axis probably results from an optical aberration caused by the inhomogeneous distribution of harmonophores within spherical starch granules^27, 44^ and by the index-mismatched interfaces found in bread dough^45^.

A threshold was set in the EF mode (Fig. 6a2–d2) to delete any noise present in the gaseous cavity and obtain a smooth outline (see “Materials and methods” section for more detail). This produced an “envelope” which overlapped the segmented starch granules from the SHG images, extending just a little beyond the outermost granules. The resultant envelope might in reality be slightly wider, especially if we consider that some of the low intensity signal near the GCW outlines was excluded by the selected threshold. This threshold adjustment is worthy of investigation in its own right, but the order of magnitude obtained was judged to be sufficiently accurate for the exploratory purposes of our study. As a precaution, though, this uncertainty was propagated in the calculations involving the envelope section (see the sensitivity analysis in “Materials and methods” section).

The sum of segmented starch pixels divided by the sum of segmented envelope pixels yielded an estimate of the starch fraction in each GCW: 61.5 ± 2.0% (GCW1); 49.3 ± 7.6% (GCW2); 56.9 ± 13.6% (GCW3); and 54.3 ± 3.7% (GCW4). The standard deviations relate to the extreme variability in the size and number of starch granules to be found in the slices evaluated at different depths within the image stacks. The high averages and dispersion of the values will be discussed further in the next section.

**Figure 7:**
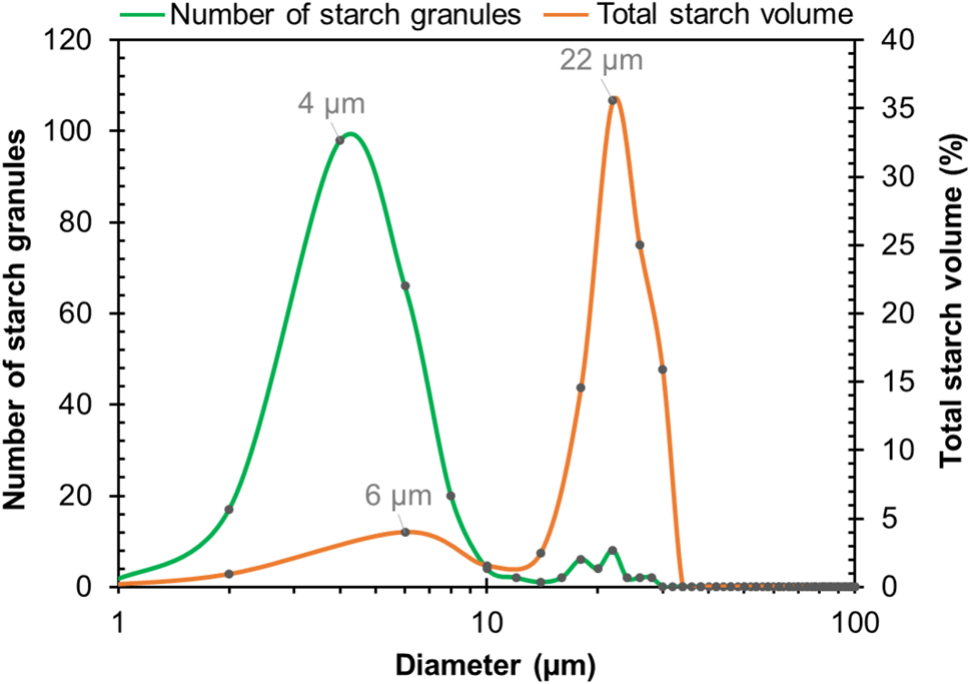
Size distribution of starch granules in GCWs; calculations used the Max-Feret diameter of each segmented starch granule in a representative slice of each GCW and the spherical assumption for granule volume (see “Materials and methods” section for details). Symbols indicate the values obtained per class (the value—either granule number or starch volume—was centered on the middle of the class); the fitting line is indicative.

The size distribution of starch granules in the GCWs was also calculated using the starch segmentation data and the results are presented in Fig. 7. Since the number of starch granules in a single GCW is very limited, all segmented starch granules from the four GCW evaluated were considered (554 in total). This quantity can still be considered low compared to those produced by other techniques used for particle size distribution, but it should be remembered that the segmentation of the starch granules was performed manually in the present study. Notwithstanding this low total, the curves obtained were relatively smooth and were hence comparable to those in the literature^38^, also classically acquired in 2D.

As expected for wheat starch, size distribution was trimodal in the GCWs observed in the present study, with peaks at around 4 µm, 6 µm and 22 µm for C-, B- and A-types, respectively. Note that in Fig. 7 both number and volume proportion (left-handed and right-handed y-axes respectively) are shown to allow these types to be distinguished, given that, for instance, C-type granules in wheat starch are present in the highest numbers but their volume proportion is negligible, while the reverse applies for A-type granules. The proportions in our samples were also roughly in line with these (see further discussion below). Peaks for A- and B-type granules were similar to those reported by Wilson et al.^38^, 6–7 and 24–25 µm respectively. The main difference lay in the peak position of the smallest granules—whereas Wilson et al.^38^ recorded a narrow peak at 1–2 µm, we encountered a wide peak at 4 µm. This could be because the detection of these granules using NLOM is defective (meaning that B-type granules exert a greater influence on size distribution and there is a shift to higher values in the peak). The same reason could explain the higher volume proportion for A-type granules (around 40% at peak, versus 6% in Wilson et al.^38^). Differences in wheat varieties and milling procedure between the studies might also explain the difference in C-type granule distributions and further investigation to differentiate between factors would be required before any clearer conclusions can be drawn about the possible limitations of NLOM.

## Discussion

In this work, multimodal acquisition combining EF and harmonic generation signals from a label-free proven bread dough was used for the first time to analyze the spatial dispersion of starch granules and gluten network in GCWs, also examining the morphology of the GCW envelope and these embedded starch granules. This innovative approach was selected as it allows the live imaging of proven dough without the need for labeling, thanks to endogenous fluorescence and harmonic generation assignable to protein and starch respectively. High autofluorescence intensity allowed imaging of the gluten phase, second harmonic generation imaged the starch granules and third harmonic generation imaged the interfaces of bread dough, while the gas cells could be detected using contrast (signal absence in these three modes). Third harmonic generation was not further analyzed, however, because of its lack of contrast in the depth of acquisition.

In contrast, second harmonic generation and endogenous fluorescent measurements demonstrated their strong potential as methods for the 3D imaging of bread dough. The dual excitation 1040 nm/1240 nm allowed to acquire SHG of whole starch granules without doing any supplementary acquisition with linear polarization. Nevertheless, a more standardized protocol was selected to be used in our main experiments, applying a single excitation wavelength (820 nm) combined with controlled linear polarization. In this way, a larger scientific community could benefit from the protocol here applied.

Through these experiments, we observed that the gluten phase appeared to twist between groups of starch granules, suggesting that in GCWs, the two phases are still interwoven with each other. This spatial organization differs from that proposed by Eliasson and Larsson^20^, which serves as a reference for the cereal chemistry community, where it is suggested that the gluten is concentrated outside and starch inside the GCWs.

Through the use of multiphoton imaging with high resolution we determined that each granule was surrounded by autofluorescent material with varying degrees of intensity. This introduces the future challenge of disentangling the contributions made by gluten and the extragranular aqueous phase to this autofluorescence. Gluten is said to envelop each starch granule at the optimal stage of mixing, while very little is known about the extragranular phase, since it appears in varying amounts depending on the water absorption of the dough’s ingredients (starch, but also arabinoxylans for instance). This contrasts with what can be assumed to be an important role in GCW stabilization^19^. The presence of gluten independently of starch was observed only in our study of strings—that is, once rupture had taken place. Indeed, once a gluten film has reached the size of starch granules it becomes instable, meaning that there is very little chance of it being observed under the microscope. This also explained a degree of blurring over the full length of GCWs whose thickness approaches that of lenticular granules (20–30 µm), such as GCW4 (Fig. 5c4). Last, the autofluorescence intensity was found to be higher and more continuous in the thinner GCWs and strings (Fig. 5). Indeed, gluten enrichment of GCWs upon stretching is to be expected due to the strain hardening mechanism, and this matter merits further investigation (here, we return to the issue of the assignment of autofluorescence but we also suggest the taking of dynamic measurements in order to monitor the concentration occurring in the autofluorescence that is suggested by our interpretation).

Starch granules were detected in SHG images independently of the other dough components thanks to the high specificity of the harmonic signal limited to the non-centrosymmetric structure of amylopectin^28^. The combination of forward and backward detection with four polarization angles allowed individual granule segmentation, paving the way for highly specific quantitative analysis of the granular phase, such as the determination of the starch fraction and granule size distribution in GCWs. It is important to emphasize once again that the combination offered by four-angle polarization provided more reliable access to the morphology and size of the starch granules, without which smaller granules (C- and even B-type) could have been missed completely. In addition, harmonic imaging has the particular quality of not being sensitive to photobleaching^24^ enabling long acquisition times without loss of signal.

Once the granules had been segmented, it was possible to observe that not all of the largest starch granules preferentially orientated their longest sides along the GCW direction of extension. This observation contrasts with the neat alignment reported by Sandstedt et al.^3^ when demonstrating the imaging of thin GCWs for the first time using optical microscopy combined with the staining of proteins. Meanwhile, it is consistent with SEM observations of the internal surfaces of gas cells where starch granules presented their largest sides perpendicular to the direction of dough extension^46^.

Starch fraction has been rarely estimated in the literature, even for bulk dough. Bloksma^47^ estimated that starch granules occupy about 60% of the dough’s volume, being this approximation based on a calculation using flour and dough composition as the reference, but very few details on the exact calculation steps were given. A similar, but more detailed approach was used by Tanner et al.^48^, who obtained values for the starch volumetric fraction in bulk dough that varied from 46 to 60%. The different values were due to variations in dough water content and in the consequent swelling of the starch through water absorption. Tanner et al.^48^ also took other factors into consideration to obtain these values, including the starch fraction in the flour, the percentage of damaged starch granules (a major influence), dough and starch densities, and the fraction of A- and B-type starch granules. The use of calculation, despite the many hypotheses it requires, can be useful to estimate the starch fraction when no other means are available. A pioneering quantitative analysis of microscopic images of dough was carried out by Mohammed et al.^49^ in 2013. They used cryo-SEM images of non-yeasted bulk dough for this purpose. However, comparison between original and treated images (both provided in the paper) clearly showed that the starch content was underestimated (many granules were not segmented, most probably due to lack of contrast), possibly explaining the low value reported for the starch fraction (45%) in their paper. Following on from this initial work by Mohamed et al.^49^, we analysed NLOM images of GCWs after manual segmentation of the starch granules to estimate the starch fraction in the GCWs. Overall, a starch fraction of 56 ± 9% was found (average value based on the individual values calculated for GCW1–4).

The high variation in these values can be explained by the particularities of each GCW analyzed. Thus, on the one hand, string shaped GCWs containing areas of gluten that were free of starch granules yielded starch fraction values from 59 to 64%; this range being consistent with Bloksma’s^47^ and Tanner’s^48^ calculations. On the other hand, in thicker GCWs, the starch granule fraction ranged from 33 to 65%. For the “strings” it is possible that, following rupture, the starch granules agglomerate at the edges. Here, the presence of some granule-free areas would be compensated by the greater fraction of granules at the edges, giving a somewhat higher average value for strings. For “thick” GCWs, the high level of variation can be explained by the variability of the presence of starch granules at different depths. Despite such variations, this order of magnitude has been estimated experimentally for GCWs for the first time (i.e. not for bulk dough^49^ and not through calculation^47, 48^ as previously described in the literature). This finding will require further exploration under different conditions (different dough preparations for instance) with a larger number of samples, but already, this order of magnitude is of utmost importance for the initialization and validation of numerical models reproducing the mechanics of GCWs, such as that described in Mohammed et al.^49^ or Dedey et al.^50^.

Our study also demonstrated that the size distributions of granules, as analyzed in GCWs from NLOM measurements, were similar to those reported for wheat flour^38^, suggesting that the extension of the dough would not involve the full segregation of certain starch types.

Because of the deformation of the granules along the z-axis (depth) that resulted from optical aberration, the entire quantitative analysis for starch was performed in 2D in the first instance. Alternative solutions could be investigated to decrease the elongation of starch granules along z-axis, such as work on refractive index matching. Image analysis in 3D will also require the setup of an automatic segmentation routine. The 3D value of our microscopic investigation of GCWs was uncompromised in the EF mode. In fact, the exploration depth of the EF (highest value of 46 µm) exceeded in 2 to 4 times the thickness of the largest granules of starch. It was found to be useful in distinguishing strings from intact walls (Fig. 6a3–d3). We can also point to the improved insights into the granular phase in GCWs gained through the quantitative analysis of NLOM images, albeit conducted in 2D. Moving beyond our focus on starch, and with room for further work to distinguish between the gluten and extra-granular phases (as suggested above), this study shows the great potential of microscopy to unravel the organization of multiple phases and components in GCWs in a quantitative manner.

## Conclusion

This study was ambitious in seeking to visualize both the granular phase and the gluten network in GCW of proven dough without labeling through the use of NLOM, and the results obtained show considerable promise. Through it, we have demonstrated for the first time that NLOM enables (i) the specific separation of starch granules from the gluten network in bread dough by using two investigative acquisition modes—TPEF for endogenous fluorescence imaging and SHG for starch granule imaging, and (ii) the non-invasive analysis of the microstructural features of bread dough and gas cell walls, thus demonstrating that this innovative method can be successfully applied in dynamic imaging. In particular, this new approach has real potential for the monitoring of GCW microstructure during proving. With the help of future work to improve the fast imaging parameters and the use of AI to facilitate the 3D segmentation of starch granules for image analysis, this is a real prospect.

## Materials and methods

**Figure 8:**
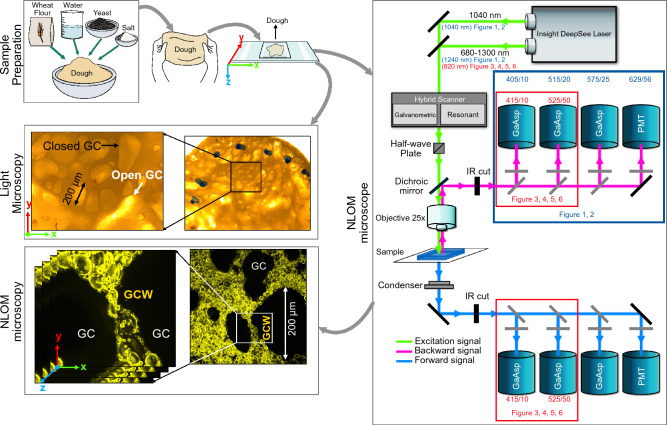
Diagram of the steps in sample preparation and analysis. *GC* gas cell, *GCW* gas cell wall. Refer to Table 1 for more information about the acquisition parameters of the NLOM microscope.

The different steps of sample preparation and NLOM analysis are illustrated in Fig. 8.

### Sample preparation

The bread dough was prepared according to the following recipe: 245.0 g of wheat flour (from Moulins Soufflet Pantin, France, type 65, 0.53% ash, 10.58% proteins, Alveolab® P/L = 0.66), 147.0 g of water, 4.9 g of salt and 2.49 g of powdered yeast (Lesaffre Saf-Instant yeast). The ingredients were mixed using an alveograph (Alveolab®, Chopin technologies, France), using the following protocol: the powders were blended for 1 min, after which the water was added and the ingredients were mixed for 1 min 30 s. The equipment was then turned off and ingredients adhering to the walls of the chamber were removed and manually mixed with a spatula. The dough was then kneaded for 10 min. After mixing, the temperature of the dough was 25 °C ± 1 °C.

Dough pieces (15 g) were sampled, placed inside graduated plastic pots, and stored overnight under refrigeration (dough core temperature was 1.0 °C ± 0.3 °C). Volume increase was less than 1.37 (+ 37%) overnight, indicating that proving was very limited because of the low temperature. The dough was transported in a cooler to the microscopy platform and kept cold once there. Very small dough samples (occupying about a quarter of the glass coverslip section once in place on the spacer) were removed regularly from the pots throughout the day of the experiment and were flattened between a glass slide and coverslip separated from each other by a 250 µm spacer (Fig. 8). A small vertical cut was made in each sample to allow gas exchange between the sample and the environment (avoiding excessive pressure from yeast activity). The slides were placed in a chamber regulated at 30 °C for about 2 h before being imaged. Advanced proving was chosen for this preliminary work in order to limit the movement of the dough during acquisition.

Using optical microscopy, two different types of gas cells could be observed in the dough sample at advanced stages of proving (Fig. 8, indicated by arrows). Cells were either closed (sphere-like) or opened (“tubular”), the latter being in direct contact with the coverslip, as could be recognized by the appearance of the coverslip, which was opaque or translucent, respectively. The former were small (approximately 200 µm) while the latter were often much larger. It is important to mention that the samples containing the GCWs selected for evaluation (GCW 1–4) were not individually assessed before the NLOM analysis—it is not therefore possible to provide their precise locations in the samples. However, in light of the size of the gas cells surrounding them (approx. 200 µm), as observed using NLOM at the highest field-of-view possible with the selected lens, we can assume that our selected GCWs were still closed and had not become elongated in the vertical direction through direct contact with the coverslip.

### Multiphotonic imaging: fluorescence and harmonic

Measurements were performed on a multiphoton microscope (A1RMP-HD, Nikon Europe B.V., Amsterdam, Netherlands) coupled with an Insight Deepsee laser (Spectra Physics, France), used in the 820–1300 nm range < 120 fs pulse width (APEX platform, INRAE/Oniris UMR 703 PAnTher Nantes, France, Center of Excellence Nikon Nantes). An auxiliary beam at 1040 nm was available in combination with the tunable output for dual wavelength excitation. For all images, an apochromat 25X MP1300 immersion objective was employed (NA 1.10, WD 2.0 mm) and distilled water was used as the immersion medium. A half-wave plate (HWP; MKS-Newport, USA) was rotated to control the laser polarization angle in the range 0°–135°. The microscope was equipped with eight Non-Descanned Detectors (NDD), three GaAsP (gallium arsenide non-descanned) and one photomultiplier tube for backward detection, with the same detectors also configured for forward detection.

The endogenous fluorescence (EF) was probed with Two Photon Excitation (TPE). SHG was detected in both backward and forward channels, whereas THG was detected only in the backward channel. Different dichroic and emission filters from Semrock were used according to the excitation wavelengths and analyzed signals (fluorescence and harmonic signals). More information on the acquisition parameters can be found in Table 1.

### Image analysis

The images that appear in the figures in this paper were treated using NiS-Elements software (5.40.01, Nikon Instruments Inc., Nikon Europe B.V.).

Segmentation was carried out using Avizo software (Thermo Fisher Scientific). Segmentation of the starch granules was performed from images combining all modes (EF and forward and backward SHG with four polarization angles). The method consisted of manually defining the edges of a given starch granule (from the SHG signal) in the x–y plane of the image by using a brush tool, where each pixel was assigned to an object (label). Each successive slice was then swept in the z-direction (depth of the image stack) so that the reconstruction of the granule could be as faithful as possible. To speed up the segmentation process, some slices were omitted, and these missing intermediate slices were automatically interpolated by Avizo (interpolate function). The sequence of operations was then repeated for another granule. Visual scrutiny of the slices while carrying out the granule segmentation made it possible to determine the effective SHG-mode depth analyzable by manual segmentation, being 15.5 µm of analyzable depth for the GCW1, 15 µm for GCW2, 7.5 µm for GCW3 and 15 µm for GCW4. The selected slices were the same considered as candidates for the 3D representation in Fig. 6. In other words, the SHG signal was acquired over a larger domain (depth), but the signal in the discarded slices was too low to offer sufficient contrast for starch granule segmentation.

For each object (granule), it was then possible to obtain individual quantitative data, such as position, volume and dimensions, using the “Label Analysis” tool from Avizo. The classic method to estimate granule size distribution^38^ retains the longest dimension for each granule identified from 2D images and, assuming the shape to be spherical, converts this dimension into granule volume in order to visualize B- and A- starch types (see “Results” section for more details about these starch types). We followed this method, using the longest dimension estimated from the Max–Feret diameter to calculate the size and volume distribution of the granules (Fig. 7—“Results” section). For this calculation, a single representative slice of each GCW was selected.

Following segmentation of the starch granules, described above, it was also possible to calculate the surface area occupied by the starch in the wall, known as the starch fraction. As in the previous analysis (granule size distribution), this calculation was performed in 2D, but repeated on five slices for each GCW instead of only one. To be comparable, these slices were of the same number (five) and positioned across the same total depth for all GCWs (6 µm). For this purpose, the number of pixels defined as “starch” were added together and this value was divided by the total number of pixels for the GCW envelope, determined by applying manual thresholding to the autofluorescence images. A variation of 40% in this thresholding, representing a total variation in GCW thickness of 3–4 µm, yielded a variation of 5% in the starch fraction. This value was lower than the variation due to the slice position (and the number of starch granules between slices). The calculation was performed on five slices for each GCW so that the variation of granule numbers in the depth direction could be taken into consideration, yielding a mean value and its standard deviation. Additionally, the first slices of each domain were discarded to avoid a border effect in the granules (where size/number of starch granules are not representative). The selected stacks for the starch fraction calculation started from different slices for each GCW.

### Supplementary Information

Supplementary Figure S1.

## Data Availability

This work is licensed under a Creative Commons Attribution 4.0 International License. Images or other third-party material appearing in this article are included in the article’s Creative Commons license, unless indicated otherwise in the credit line; where any material is not included under the Creative Commons license, users will need to obtain permission from the license holder to reproduce the material. To view a copy of this license, visit http://creativecommons.org/licenses/by/4.0/. If further data from this study are desired, please contact Nanci Castanha (nanci.castanha-da-silva@inrae.fr) or Tiphaine Lucas (tiphaine.lucas@inrae.fr).

## Abbreviations

EF: Endogenous fluorescence
GCW: Gas cell wall
LM: Light microscopy
NLOM: Nonlinear optical microscopy
SHG: Second harmonic generation
THG: Third harmonic generation
TPEF: Two photon excitation fluorescence
