# Supplementary material for: Multiphoton microscopy is a nondestructive label-free approach to investigate the 3D structure of gas cell walls in bread dough
Source: Sci Rep. 2023 Aug 26;13:13971. doi: 10.1038/s41598-023-39797-w (PMC10460382; doi:10.1038/s41598-023-39797-w)
Supplement: Supplementary file 1 — Supplementary Figure S1. [file 41598_2023_39797_MOESM1_ESM.docx]

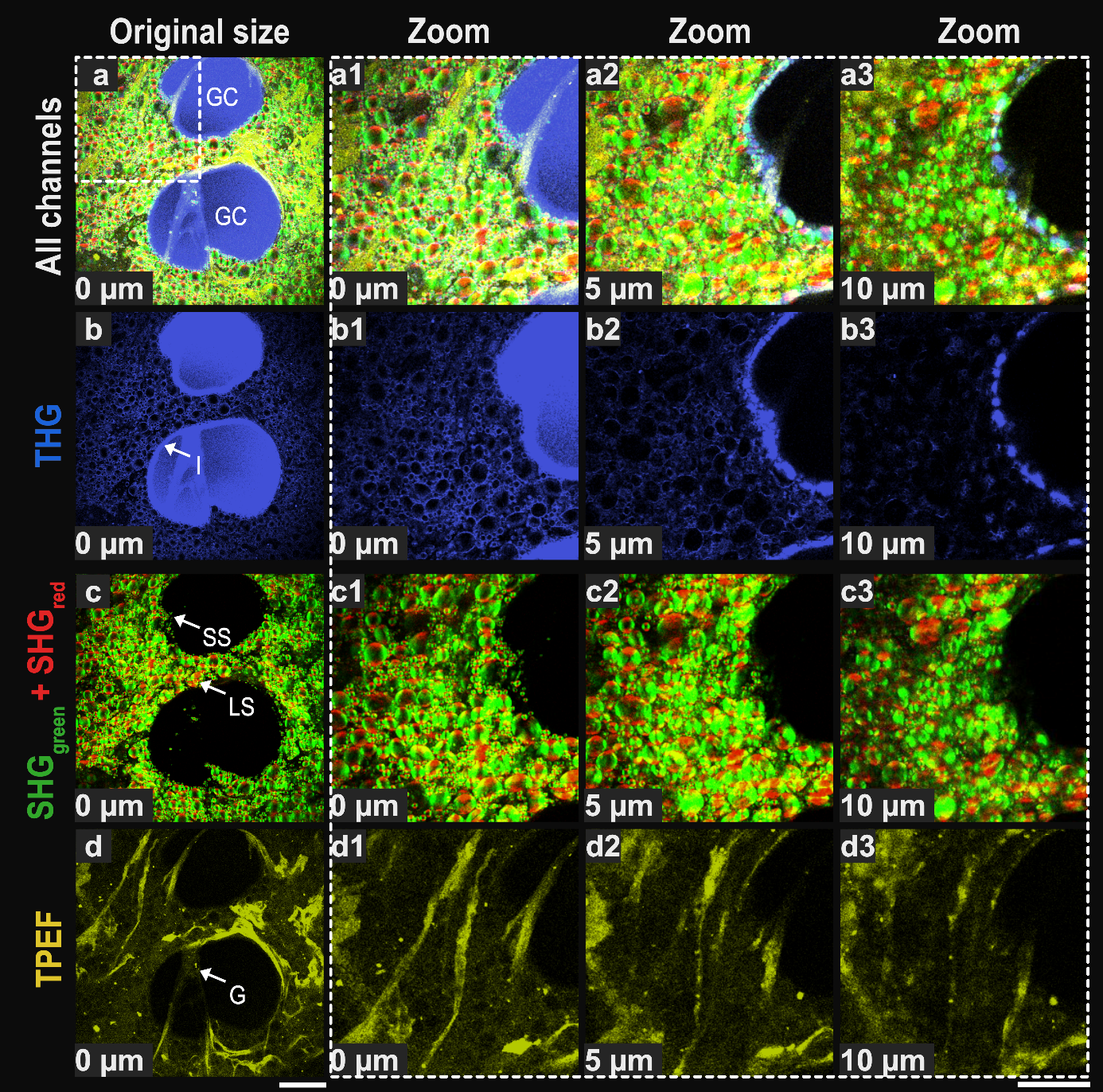


Figure S1. Second harmonic generation (SHG) and third harmonic generation (THG) combined with endogenous fluorescence (EF) to image starch granules, gluten network, and gas-dough interface in label-free bread dough (bulk) at the end of proving. Region of Interest (ROI) is delimited by the dotted white square in a), and can be observed at different depths for each channel (z-stack 0, 5 and 10 µm). a) combination of the three modes, all channels (THG, SHG, EF); b) THG in blue (1240 nm); c) SHG in green and red in dual excitation: 1040 nm (green)/ 1240 nm (red); d) EF in yellow (1040 nm). GC = gas cell; I = interface; SS = small starch granules; LS = large starch granules; G = gluten. Scale bar = 100 µm (original size); 50 µm (ROI).

LSG

VSSG
